# A compatible model for aboveground biomass of individual culms in *Dendrocalamus brandisii* plantations, Hainan, China

**DOI:** 10.3389/fpls.2026.1780589

**Published:** 2026-05-22

**Authors:** Bin Wu, John L. Innes, Yancui Ding, Qian Li, Shixin Deng, Renyi Gui

**Affiliations:** 1 Bamboo Industry Institute, Zhejiang A&F University, Hangzhou, China; 2 College of Mathematics and Computer Science, Zhejiang A&F University, Hangzhou, China; 3 Department of Forest Resources Management, University of British Columbia, Vancouver, BC, Canada; 4 National Key Laboratory for Development and Utilization of Forest Food Resources, Zhejiang A&F University, Hangzhou, China

**Keywords:** *Dendrocalamus brandisii*, biomass allocation, bootstrap, compatible model, seemingly unrelated regression

## Abstract

**Introduction:**

*Dendrocalamus brandisii* is a high-yielding bamboo species valued for both its carbon-sequestration capacity and its economic and ecological benefits. Despite this, quantitative information on how its above-ground biomass (AGB) is accumulated, partitioned, and scaled across different climatic zones is still scarce.

**Methods:**

We addressed this knowledge gap using a destructive sample of 45 culms (2- and 3-year old culms) harvested from a managed plantation in Sanya, Hainan, China. Two modeling strategies were compared: (i) independent models fitted by weighted non-linear least squares and (ii) compatible models based on Seemingly Unrelated Regression (SUR) that guarantee additivity among culm, branch, and leaf biomass. Predictor variables were diameter at breast height (DBH), total height (H), and a combined variable (DBH)²H. Model performance was evaluated using 1,000 bootstrap iterations with out-of-bag (OOB) predictions, and final model parameters were estimated from the full dataset.

**Results and discussion:**

2-year-old culms were roughly twice as large in DBH and H, and contained fourfold more AGB than 3-year-old culms. Independent models demonstrated that DBH was the most effective singular predictor for each component and AGB. Compatible SUR models achieved similar accuracy (R² > 0.9 for AGB and culms, > 0.7 for leaves, > 0.5 for branches) while reducing the discrepancy between summed components and total AGB to virtually zero. We developed the first SUR-based compatible biomass models for *D. brandisii* in Sanya City, reducing carbon-accounting discrepancies between component and total aboveground biomass (AGB) to near-zero, which could support accurate measurement of the bamboo-forest carbon sink. The developed system could be directly applied to Tier-3 carbon monitoring and climate-adaptive management of *D. brandisii* plantations under similar edaphoclimatic conditions.

## 1 Introduction

Forests play an important part in global carbon sequestration and storage. Consequently, countries worldwide place great importance on monitoring and assessing forest biomass (Zhou et al., 2023). In subtropical and tropical regions, bamboos form an important component of natural forests, and their role in carbon sequestration and storage is increasingly recognized. Because of their many uses and environmental services, including carbon sequestration and storage, a number of different species of bamboo are increasingly being grown in plantations. Numerous attempts have been made to estimate the biomass of bamboo and other forests. Satellite data from WorldView, MODIS, and Landsat, as well as UAV-LiDAR data, have been used in conjunction with machine learning methods, including CNN, Random Forest, and Cubist, to develop high-precision biomass estimating models (Dong et al., 2020; Wi et al., 2017; Zhang et al., 2019; Li et al., 2018; Li et al., 2024a; Esteban et al., 2019; Zhang et al., 2024). Although remote sensing is widely used to estimate aboveground biomass in bamboo forests, acquiring high-precision data is costly. Free datasets are limited by their temporal and spatial resolutions. Furthermore, remote sensing data still require ground-based measurements for calibration. Consequently, empirical biomass models remain practical and cost-effective tools, particularly for the management of plantation forests (Wang et al., 2025, Wang et al., 2016; Wu et al., 2019; Yen and Lee, 2011).

A biomass model is a statistical model that uses mathematical methods to show how the biomass of trees or forest stands is associated with variables that can be readily measured, including diameter at breast height, plant height, volume, and others. Its accuracy and reliability as a statistical tool for measuring the link between biomass and plant variables have a direct impact on how accurately we can estimate forest carbon storage (Ballesteros-Possu et al., 2016). Early biomass models mostly used polynomial representations of linear models to clarify the correlation between biomass and independent variables (Ballesteros-Possu et al., 2016). Even though polynomial models can fit the biomass data to some extent, they have clear limits (Johnston, 1977). As research progressed, the potential for a more nonlinear relationship between biomass and tree variables was increasingly recognized. Baskerville (1972) systematically explained the guidelines and methods for using logarithmic regression to figure out how much plant biomass there is, as did later studies (e.g., Bates and Watts, 1988; Beauchamp and Olson, 1973). Logarithmic transformation can be employed to accommodate nonlinear models, such as the allometric model; however, it is susceptible to bias (Van Hooser and Van Pelt, 1985, p. 73). With improvements in computer technology and the growth of computing power, the use of nonlinear models in forestry has become more common (Melo et al., 2017). The parameters of nonlinear models often have clear biological meaning and can better show how trees grow (Melo et al., 2017; Lima et al., 2019). An allometric growth equation, also called a power function model or total product model, is one of the most common nonlinear model forms used in biomass modeling. It shows how biomass and morphological variables are related (Melo et al., 2017; Parresol, 1999). Such models are widely used to estimate aboveground biomass, and they have worked well in many places (Yuen et al., 2017).

When modeling individual biomass, the additive problem frequently occurs, necessitating that the cumulative predicted values of each component (including stem, branches, leaves, and roots) must equal the predicted value of the total biomass. However, biomass components are usually modeled separately, and parameters that are estimated separately cannot ensure that the total and the parts are logically consistent (Affleck and Diéguez-Aranda, 2016; Affleck, 2015; Mao et al., 2022). Consequently, a combination of total and component biomass models can be regarded as a model system for resolving the additive problem. The corresponding methods include the generalized method of moments (GMM), proportional adjustment and nonlinear joint estimation, seemingly unrelated regression (SUR), and non-linear seemingly unrelated regression (NSUR) methods (Meng et al., 2021;Behling et al., 2018; Parresol, 1999). Among them, seemingly unrelated regression (SUR) is a typical representative method (Shahbaznezhad et al., 2021; Huy et al., 2019a; Parresol, 2001) that is widely used in biomass models to ensure the additivity of each component (Pérez-Cruzado and Rodríguez-Soalleiro, 2011; Cunha Neto et al., 2022). Its core idea is that the total biomass should be equal to the sum of the biomass of each component (Bronisz and Mehtätalo, 2020). The SUR method ensures that total biomass equals the sum of its parts by simultaneously estimating the equation system. This approach addresses heteroscedasticity and resolves the singularity problem of the cross-equation covariance matrix (Meng et al., 2021, Meng et al., 2019).

*D. brandisii* is an economically important bamboo species found in the wet evergreen tropical forests of Southeast Asia, where it occurs at altitudes up to 1300 m asl. Its natural range extends from northeastern India through northern and eastern Myanmar, into northern Thailand, Laos, northern Cambodia, northern Vietnam, and southwest China. In China, it is widely distributed in western and southern Yunnan, occurring naturally at altitudes between 380 and 1900 m (Kunming Institute of Botany, Chinese Academy of Sciences, 2003; Hui et al., 2019; Tian and Zhang, 2022). It is one of the largest clumping bamboos in the world, with its culms attaining heights of up to 33 to 36 m and diameters up to 20 cm under ideal growing conditions. However, its size diminishes toward the edges of its natural range and at higher altitudes. Because of its large size and rapid growth, it is frequently used as a plantation species. Under suitable growing conditions, its carbon accumulation rate is comparable to or even higher than that of fast-growing tree plantations (Sohel et al., 2015). *D. brandisii* is favored as a plantation species as it has multiple functions. Its culms are used in construction or are broken down for engineered bamboo products (e.g., Kelkar et al., 2020), and the sheaths offer potential for paper-making (Zhan et al., 2017). Its shoots are widely harvested and have a tender texture, sweet flavor, and versatile culinary applications (stewing, stir-frying, or fresh consumption). This makes the shoots a high-value economic food resource (Hui et al., 2019; Xue et al., 1995).

Research on *D. brandisii* has primarily focused on traditional aspects of bamboo cultivation, such as its biological characteristics (Huang et al., 2020a), and the effects of management measures (Yan et al., 2024; Bahru and Ding, 2020) and site conditions (Zhou et al., 2015; Liu et al., 2021) on its growth. There has also been research on the factors that encourage a high yield of shoots and on the use of technology to encourage earlier sprouting of shoots (Chen, 2021; Wang et al., 2015; Shi et al., 2011). However, there has been very little research on the aboveground biomass of *D. brandisii*, and the work that has been done was completed in southwest China, in Yunnan Province (Ji et al., 2015; Wang et al., 2025). Work on other bamboo species, such as *Phyllostachys edulis* (Zheng et al., 2024), *Pleioblastus amarus* (Zuo et al., 2024), and *Yushania niitakayamensis* (Wu and Kao, 2021), has demonstrated that phenotypic variation in bamboos can be considerable. However, there is a marked lack of knowledge concerning the phenotypic variation of most bamboo species, including the key species used in plantations. As a result, building biomass models for individual species in a specific location remains extremely important for enhancing the accuracy of regional biomass estimates for a particular species, especially when grown in plantations (Zhao et al., 2019a). Biomass models could provide farmers with scientifically informed management strategies (Chen et al., 2005) that would enable them to assess carbon sequestration capacity accurately, and which would enhance the economic and ecological value of forests (Ouyang et al., 2022; Jyoti Nath et al., 2009).

In this study, we examined *D. brandisii* introduced in Sanya, Hainan, China in 2021, to quantify aboveground biomass and its component allocation under an oceanic-influenced tropical monsoon climate. Site conditions were considered as background environmental covariates rather than explicit causal drivers, providing contextual information for model development rather than being formally tested as independent determinants of biomass variation. We aimed to evaluate different types of biomass models to identify the optimal formulations for each component and to develop a compatible system capable of simultaneously estimating individual components and total aboveground biomass. Our goal was to provide a theoretical basis for carbon stock accounting, efficient resource utilization, and the sustainable, high-yield management of *D. brandisii* plantations.

## 2 Materials and methods

### 2.1 Study area

**Figure 1:**
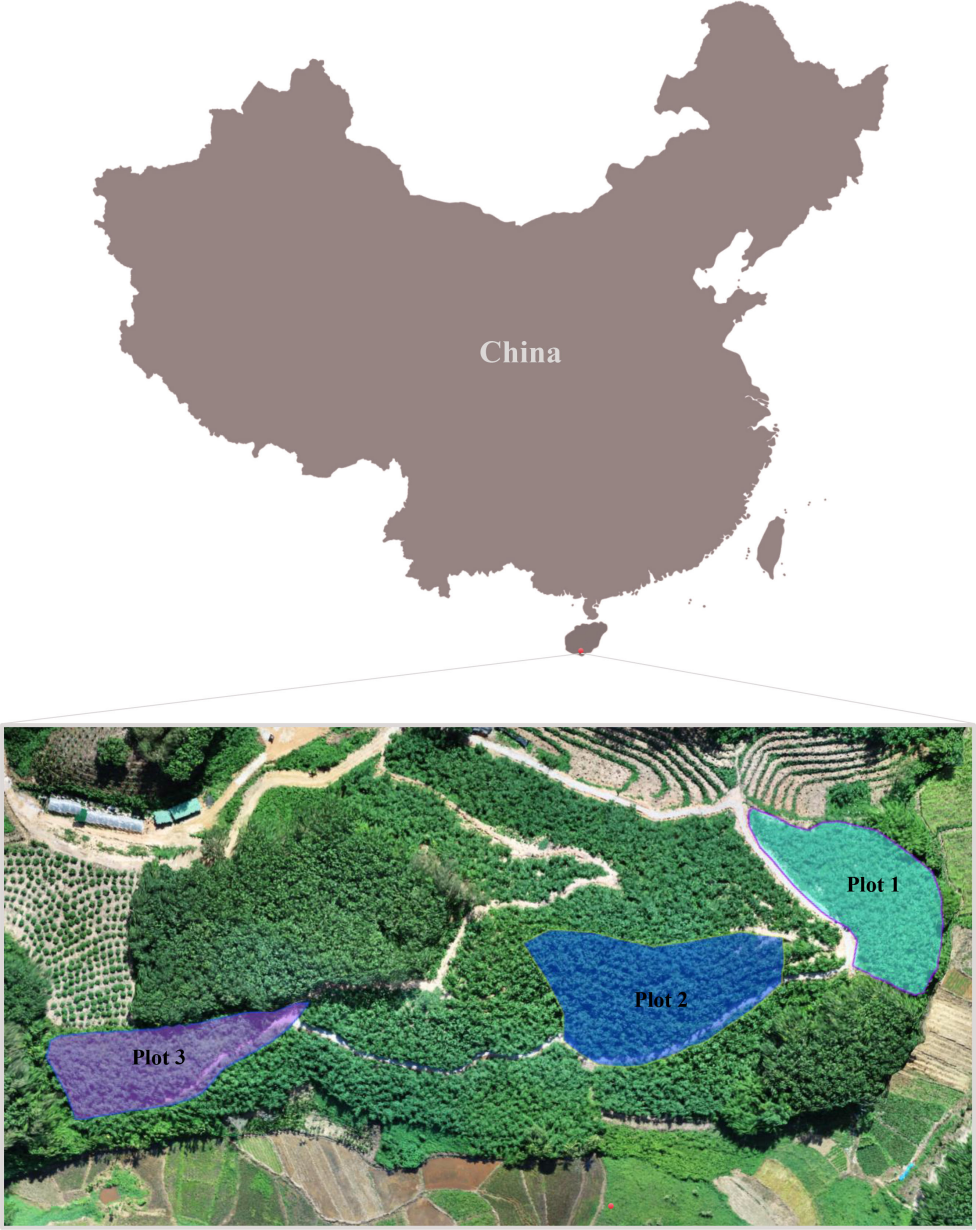
The study area is located in Sanya City, Hainan Province, China.

The research region was located in Sanya City, Hainan Province (**Figure 1**), at 108°37’~111°03’ East longitude and 18°10’~20°10’ North latitude. This location is in the oceanic-influenced tropical monsoon climate zone (Zhao et al., 2023), with an average annual temperature of 25.6 °C (Zhu et al., 2024). The average temperature in the hottest month, June, is 29.7 °C, while in the coldest month, January, it is 22.4 °C (Zhu et al., 2025). The annual average relative humidity is 85.3% (Xu et al., 2018). The annual sunshine duration ranges from 1,828 to 2,811 hours, while the average annual precipitation ranges from 941 to 2388 mm (Zhao et al., 2023). The research region lies at 168–200 m a.s.l., the soil is classified as lateritic red soil, and the previous land use was a rubber plantation. Although outside its natural range, *D. brandisii* grows well under these conditions.

The three experimental plots were in Tianya District, Sanya City, Hainan Province. Plot 1, consisting of 60 planted mother bamboo clumps, was established on a semi-sunny slope (azimuth 45°–135°) with a slope angle of 35°. In contrast, Plot 2 (70 clumps; 25°slope) and Plot 3 (60 clumps; 10°slope) were both located on semi-shaded slopes (azimuth 225°–315°). Due to the limited number of plots and the absence of a complete factorial design, the analysis of site factors primarily relied on descriptive statistics and trend observations, resulting in limited capacity for statistical inference. The number of culms per clump in each of the three plots is presented in **Supplementary Table 1**.

### 2.2 Data collection

As the *D. brandisii* plantation was established in January 2021, the bamboo culms were 1–3 years old at the time of harvesting (March – August 2024). Accordingly, the culms that emerged in the 2021 growing season were three years old at the time of harvesting, and those that emerged in 2022 were two years old. One-year-old individuals were excluded from sampling due to the absence of branches and the presence of only a few leaves. Two seasoned investigators independently identified the age (A, in years) of each sample culm based on its morphological traits by evaluating the retention status of sheaths, stalk color and wax powder, the degree of branching development, and node characteristics (refer to **Supplementary Table 2** in the **Supporting Information**) (Yebeyen et al., 2022; Zhang et al., 2020; Huy et al., 2013). A total of 45 healthy, two- and three-year-old *D. brandisii* culms were randomly selected from the experimental sites (Yan et al., 2008). All culms came from clumps that were pest- and disease-free.

The canopy of each sample stem was divided into three layers: upper, middle, and lower. In each layer, two to five leaves and one to three branches were randomly selected from five equally spaced directions starting from true north as subsamples and weighed for fresh weight. Each sampled stem was cut at ground level. Diameter at breast height (DBH) and height (H) were measured in the field. Each culm was split into three parts based on its height. A subsample of about 10 to 20 cm was taken from each part and weighed to find the fresh weight. The fresh weights of the remainder of the culm, branches, and leaves were measured, and these values were added to the fresh weights of their respective subsamples to obtain the total fresh weight of the stem. Subsequently, the subsamples of culms, branches, and leaves were oven-dried to constant weight (Mao et al., 2022; Wang et al., 2021a; Zhang, 2013). The dry-to-fresh weight ratio of each subsample was calculated and applied to the total fresh weight of culms, branches, and leaves, thereby estimating the dry weight of each component per sample stem: culms (Bcu, kg stem⁻¹), branches (Bbr, kg stem⁻¹), and leaves (Ble, kg stem⁻¹). The total aboveground biomass (AGB, kg stem⁻¹) was determined as Bcu+ Bbr+ Ble (Huy et al., 2019a).

### 2.3 Model development

#### 2.3.1 Independent biomass model development

A power function is often used to predict the biomass of single standing trees (Basuki et al., 2009; Chave et al., 2014; Huy et al., 2016; Huy et al., 2019a). DBH, H, (DBH)^2^H, and Age are commonly used as the independent variables in the model. For instance, Wang et al. (2021a) and Juan et al. (2024) utilized DBH as an independent variable to construct regression models, while Wi et al. (2017) and Huy et al. (2019a) combined DBH and H in biomass regression modeling. Additionally, (DBH)^2^H has been introduced for biomass model development (Sohel et al., 2015). Stem age has been introduced as an additional variable (Chantarat et al., 2023). The power-law relationship remains one of the most widely used models for estimating the aboveground biomass (AGB) and its components in various bamboo species (Yuen et al., 2017).

**Figure 2:**
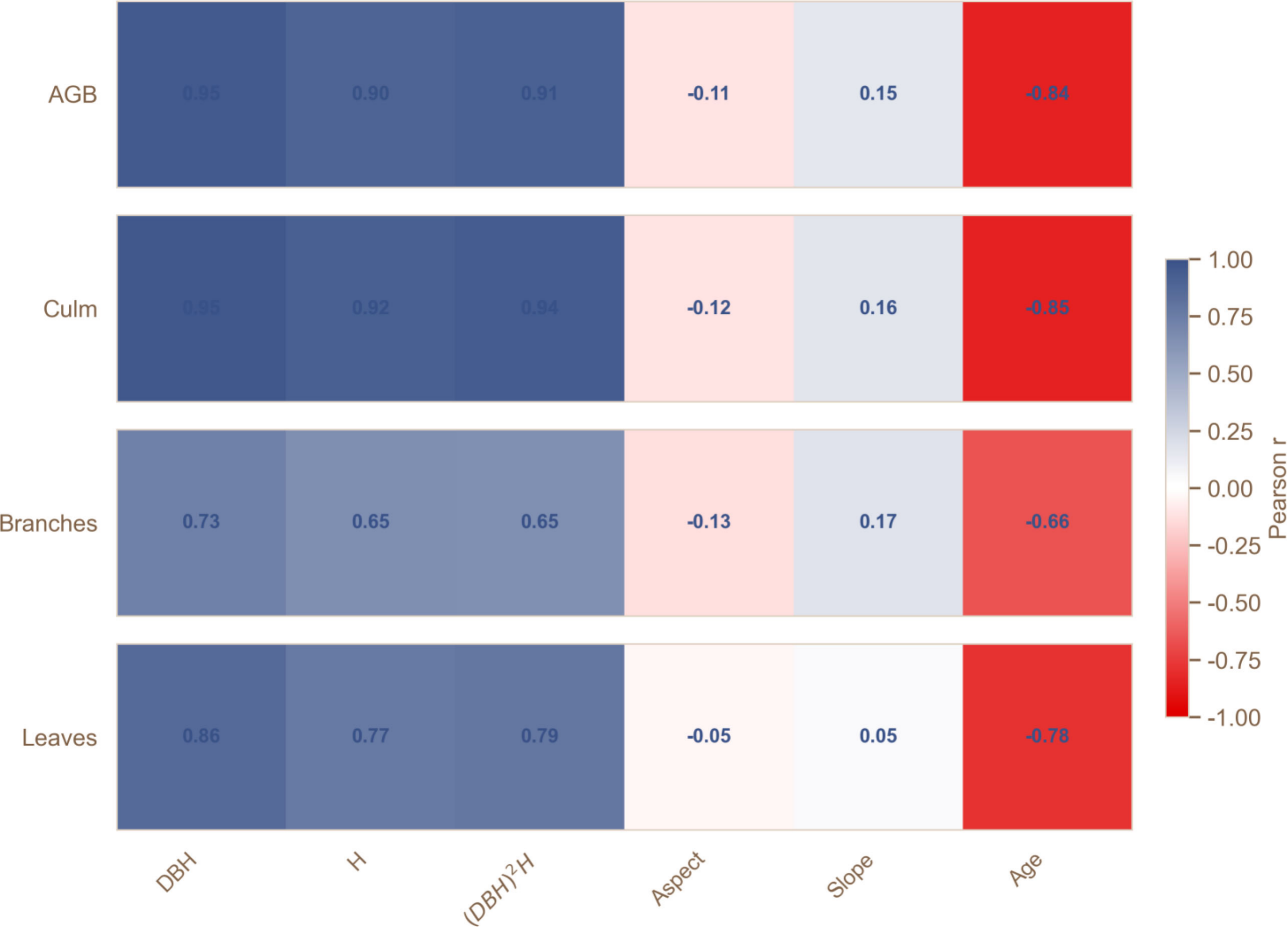
Correlation matrix between each biomass component and the predictor variables.

The research indicates that the residual variance increases in a funnel shape as the DBH (diameter at breast height) increases, indicating significant heteroscedasticity (Picard et al., 2012).The use of 1/(DBH)^2^ as the weight variable (Dong et al., 2018; Dutcă et al., 2021), effectively stabilizes the residual variance (the residual plot tends to be a horizontal band-like distribution). Other evaluation indicators include AICc, Adj R^2^, RMSE, and Bias (%). We used a power function as the basic model, and independent models of each component and aboveground biomass were developed using DBH, H, or (DBH)²H separately as alternative single predictors. **Figure 2** shows the correlation coefficients between the biomass of each component and DBH, H, or (DBH)²H. A weighted nonlinear fit was used to account for heteroscedasticity in the residuals (Huy et al., 2019a), and to compare and select the best predictor(s) for each biomass component and AGB independently (Pinheiro et al., 2018; Huy et al., 2019a). The one-dimensional model for each component and aboveground biomass is shown in Equation (1) (Liang et al., 2022):

(1) $$y_{i}=a\cdot x_{i}^{b\;}+{\text{ ϵ}}_{\text{i}}$$

where:

$y_{i}$ is the $B_{cu}$, $B_{br}$, and $B_{le}$, or AGB in kg for the i^th^ sample;

$x_{i}$ is the DBH(cm), H(m) or (DBH)^2^H for the i^th^ sample’s stem;

a, b are the parameters of the model; and.

${\text{ϵ}}_{\text{i}}$ are the errors in the equations of each biomass.

#### 2.3.2 Development of the simultaneous model system

In previous studies, when component biomass models and the AGB model were fitted independently, there were discrepancies between the aboveground biomass estimates calculated from the component models and those obtained from the independently developed AGB model (Sanquetta et al., 2015; Poudel and Temesgen, 2016; Gonzalez-Benecke et al., 2018). This limitation can be resolved by establishing a compatible biomass model system using the Seemingly Unrelated Regressions (SUR) method, which enables the simultaneous estimation of component biomass and AGB (Huy et al., 2019b; Parresol, 2001; Poudel and Temesgen, 2016; Kralicek et al., 2017).

In this research, the biomass compatibility model system was developed by combining the Direct Total-Control Nonlinear SUR with the Generalized Least Squares (GLS) method. This approach first set the aboveground biomass equation and then controlled it. After that, the equations for each component were found by using constraints or breaking them down. This made sure that the sum in the structure was consistent, that the sum of the biomass of each component was also consistent with the total aboveground biomass, and that the model residuals were not too different from each other (Chen et al., 2020; Fu et al., 2014; Tang and Li, 2002). The additivity constraint is the most important part of the compatible model system. This means that the predicted aboveground biomass must be exactly equal to the sum of the predicted culm, branch, and leaf biomasses ( $\hat{AGB}=\;\hat{B_{cu}}+\;\hat{B_{br}}\;+\hat{B_{le}}$). This constraint guarantees biological rationality and logical consistency in biomass estimation, which independent models cannot ensure (Nord-Larsen et al., 2017; Chen et al., 2023).

The compatible biomass model system is shown in Equations 2–5 (Poudel and Temesgen, 2016; Parresol, 2001):

(2) $$B_{cu}\;\;=\;\;a_{1}\cdot {x_{1}}^{b_{1}\;}\;+{\text{ϵ}}_{1}\;$$

(3) $$B_{br}\;\;=\;\;a_{2}\cdot {x_{2}}^{b_{2}\;}\;+{\text{ϵ}}_{2}\;$$

(4) $$B_{le}\;\;\;=\;\;a_{3}\cdot {x_{3}}^{b_{3}\;}{+\text{ϵ}}_{3}\;$$

(5) $$AGB=B_{cu}+B_{br}+B_{le}+\text{ϵ}$$

where $B_{cu}$, $B_{br}$, and $B_{le}$ are the biomass of culm, branch, and leaf, respectively;

$a_{i}$, $b_{i}$ are the parameters of the model (i = 1, 2, 3 for culm, branches, and leaves, respectively);

$x_{i}$ is the DBH(cm), H(m) or (DBH)^2^H for the i^th^ sample’s stem, and the weighting function is $1/x_{i}^{2}$;

AGB is the above-ground biomass, and ϵ is the error term.

### 2.4 Model selection and evaluation

Bootstrapping is a resampling method proposed by Efron (1979). It involves sampling with replacement, with the resample size equal to that of the original dataset (Efron, 1979; Myers, 1988; Crainiceanu and Crainiceanu, 2020). This approach is particularly suitable for model validation with small sample sizes (Dilsha and Kiruthika, 2014) and for assessing the accuracy of nonlinear models (Wang and Li, 2021).

The model structure was selected using a combination of the corrected Akaike Information Criterion (AICc) and bootstrap-derived predictive metrics. AICc was first applied to identify plausible candidate models for individual biomass components and aboveground biomass (AGB), after which predictive performance was evaluated using bootstrap-based R², root mean square error (RMSE), mean absolute error (MAE), and bias (%) to determine the optimal model (Vanclay, 1994; Cai et al., 2017; Huy et al., 2019b). Model performance was assessed using a bootstrap resampling approach with 1,000 iterations combined with out-of-bag (OOB) prediction, while final model parameters were estimated using the full dataset. All analyses were conducted with a fixed random seed to ensure reproducibility.

The mathematical formulas of these evaluation indicators are shown in Equations 6–12:

1. Coefficient of Determination(R-squared: R²).

(6) $${\text{R}}^{2}=1-\frac{\sum_{i=1}^{n}{\left(y_{i}-{\hat{\text{y}}}_{i}\right)}^{2}}{\sum_{i=1}^{n}{\left(y_{i}-{\hat{\text{y}}}_{i}\right)}^{2}}$$

where

${\text{y}}_{i}$ is the actual observed values;

${\hat{y}}_{i}$ is the predicted values; and.

$\bar{y}$ is the mean of the observed values.

2. Adjusted coefficient of determination (Adjusted R-squared: Adj R^2^).

(7) $$\text{Adj}\;{\text{R}}^{2}=1-(1-{\text{R}}^{2})\cdot \frac{\text{n}-1}{\text{n}-\text{p}-1}$$

where

R^²^ is the coefficient of determination;

p is the independent variables number; and.

n is the sample size.

3. Root Mean Square Error (RMSE).

(8) $$\text{RMSE}=\sqrt{\frac{1}{\text{n}}\sum_{\text{i}=1}^{\text{n}}{\left({\text{y}}_{\text{i}}-{\hat{\text{y}}}_{\text{i}}\right)}^{2}}$$

where

y_i_ is the actual observed values;

$\bar{\text{y}}$i is the predicted values; and.

n is the sample size.

4. Mean Absolute Error (MAE).

(9) $$\text{MAE}=\frac{1}{\text{n}}\sum_{\text{i}=1}^{\text{n}}|{\text{y}}_{\text{i}}-{\hat{\text{y}}}_{\text{i}}|$$

where

y_i_ is the actual observed value;

${\hat{\text{y}}}_{i}$ is the predicted value; and.

n is the sample size.

5. Akaike Information Criterion (AIC).

(10) $$\text{AIC}=\;2\text{k}+\text{n}[\text{ln}2\text{π}\frac{\sum_{\text{i}=1}^{\text{n}}{\left({\text{y}}_{\text{i}}-{\hat{\text{y}}}_{\text{i}}\right)}^{2}}{\text{n}}+1]$$

where

k is the number of parameters;

n is the sample size;

$y_{i}$ is the actual observed value; and.

${\hat{\text{y}}}_{i}$ is the predicted value.

6. Akaike Information Criterion Corrected (AICc).

(11) $$\text{AICc}=\text{AIC}+\frac{2\text{k}(\text{k}+1)}{\text{n}-\text{k}-1}$$

where

k is the number of parameters; and.

n is the sample size.

7. Bias(%).

(12) $$\text{Bias}(\%)=\frac{100}{\text{n}}\sum_{\text{i}=1}^{\text{n}}\frac{{\text{y}}_{\text{i}}-{\hat{\text{y}}}_{\text{i}}}{{\text{y}}_{\text{i}}}$$

where

y_i_ is the actual observed value;

${\hat{\text{y}}}_{i}$ is the predicted value; and.

n is the sample size.

### 2.5 Analysis

We used Excel to examine the data of each component (Neyeloff et al., 2012). SPSS 20.0 was used for some statistical analyses (Wang et al., 2021a). Model estimation, predictive performance evaluation, and data visualization for each biomass component and aboveground biomass were conducted using Python 3.11.

## 3 Results

### 3.1 Aboveground biomass composition and allocation patterns

**Figure 3:**
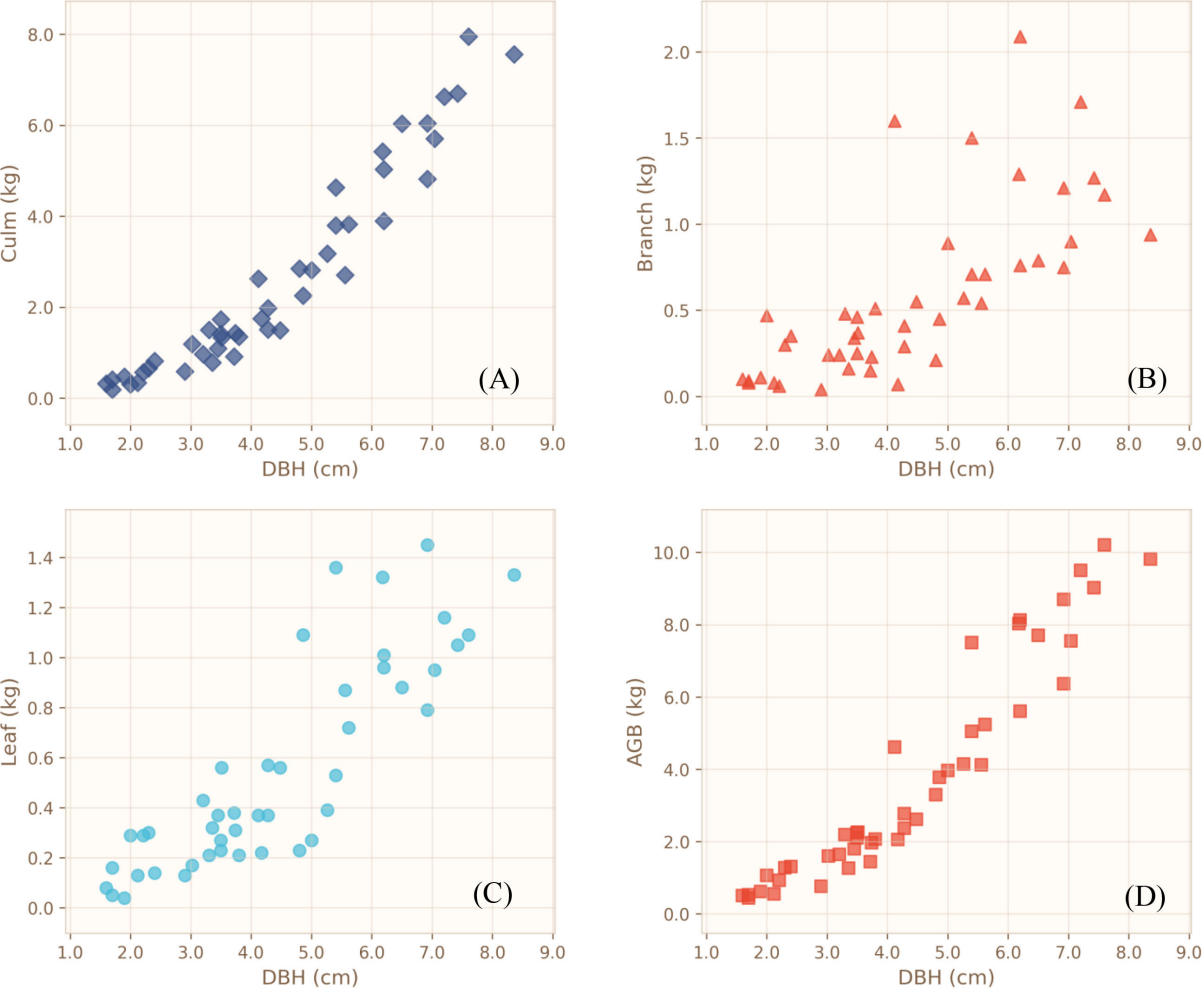
Scatterplots showing the relationships between DBH and the different biomass components. **(A)** Culm biomass; **(B)** Branch biomass; **(C)** Leaf biomass; **(D)** Aboveground biomass.

**Figure 4:**
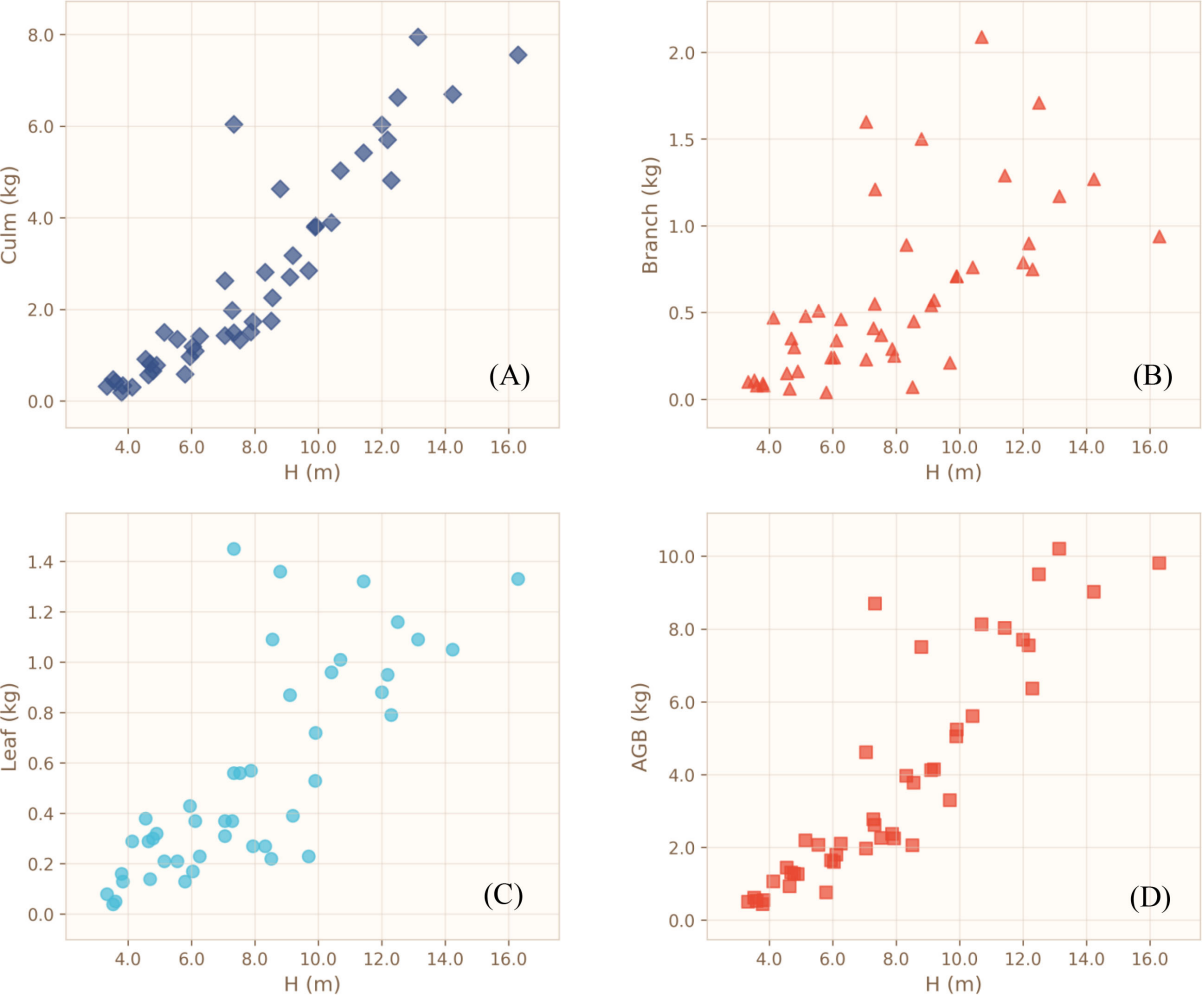
Scatterplots showing the relationships between height **(H)** and the different biomass components. **(A)** Culm biomass; **(B)** Branch biomass; **(C)** Leaf biomass; **(D)** Aboveground biomass.

**Figure 5:**
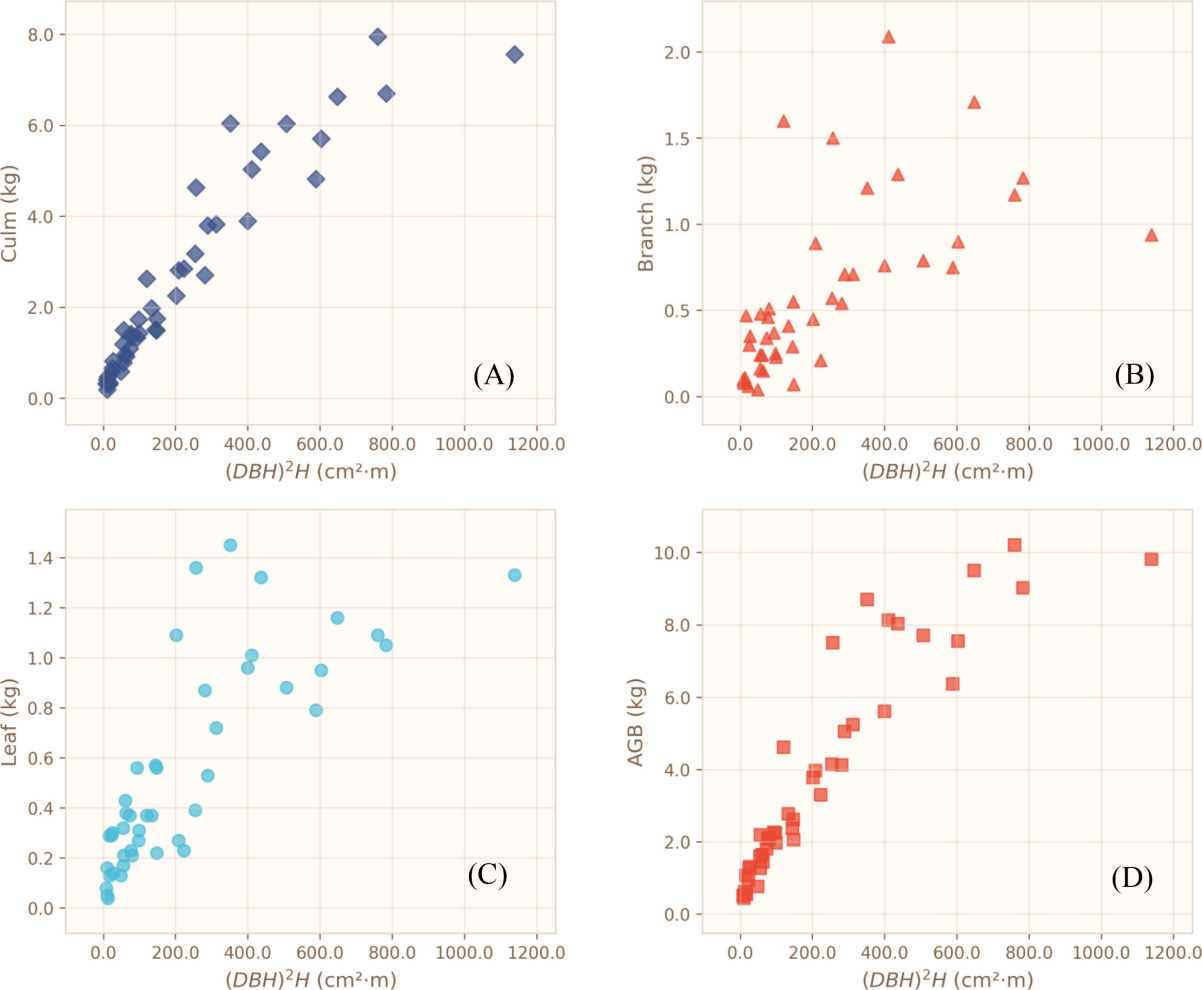
Scatterplots showing the relationships between the combined variable (DBH)²H = (DBH)² **(cm)** × H **(m)** and the different biomass components. **(A)** Culm biomass; **(B)** Branch biomass; **(C)** Leaf biomass; **(D)** Aboveground biomass.

Descriptive statistics for diameter at breast height (DBH), height (H), fresh weight, biomass, and moisture content of each biomass component are summarized in **Table 1**. Culm biomass ranged from 0.19 to 7.95 kg stem⁻¹, branch biomass from 0.04 to 2.09 kg stem⁻¹, and leaf biomass from 0.04 to 1.45 kg stem⁻¹. Scatterplots of culm biomass (Bcu), branch biomass (Bbr), leaf biomass (Ble), and aboveground biomass (AGB) against DBH, height, and the composite variable (DBH)²H are illustrated in **Figures 3**–**5**. Across all sampled individuals, culm biomass constituted the dominant fraction of aboveground biomass, with a mean value of 2.66 kg stem⁻¹, accounting for 67.40% of AGB. Branch and leaf biomasses were similar in magnitude, averaging 0.59 kg stem⁻¹ (16.25%) and 0.55 kg stem⁻¹ (16.35%) of AGB, respectively, resulting in an approximate culm-to-branch-to-leaf biomass ratio of 4:1:1. Noticeable variation in biomass distribution was detected among plots. However, given the potential interplay of soil properties, topography, microclimate, and other unmeasured site heterogeneities, all plot-level differences are presented as observational patterns without inferential interpretation.

**Table 1:** Characteristics of the 45 sampled *Dendrocalamus brandisii*.

Indicator	Mean value	Max value	Min value	Standard deviation
DBH (cm)	4.42	8.36	1.60	1.84
H (m)	7.85	16.30	3.34	3.19
Culm Fresh Weight (kg)	7.23	22.95	0.75	5.97
Culm Biomass (kg)	2.66	7.95	0.19	2.22
Culm Moisture Content (%)	62.79	78.80	45.68	8.55
Branch Fresh Weight (kg)	1.79	4.85	0.15	1.35
Branch Biomass (kg)	0.59	2.09	0.04	0.50
Branch Moisture Content (%)	67.14	88.30	44.16	12.65
Leaf Fresh Weight (kg)	1.23	3.44	0.12	0.89
Leaf Biomass (kg)	0.55	1.45	0.04	0.41
Leaf Moisture Content (%)	56.30	69.38	41.18	6.46
Aboveground Total Fresh Weight (kg)	10.25	30.05	1.03	7.96
Aboveground Biomass (kg)	3.79	10.21	0.44	3.00

Observed variation in growth traits and biomass allocation was strongly associated with stem age, slope gradient, and topographic aspect (**Table 2**). The sample encompassed individuals distributed across three slope classes (10°, 25°, and 35°) and two aspect conditions (semi-sunny and semi-shaded). Clear morphological and biomass discrepancies were observed between two-year-old and three-year-old stems. Specifically, two-year-old stems yielded a mean DBH of 6.23 cm and a mean height of 10.84 m, which were 102% and 91% greater than the values of 3.09 cm and 5.67 m recorded for three-year-old stems, respectively. Correspondingly, aboveground biomass in two-year-old stems reached 6.72 kg stem⁻¹, nearly four times that of three-year-old stems (1.65 kg stem⁻¹), with culm, branch, and leaf biomass showing 3.5-fold, 2.1-fold, and 2.3-fold increases, respectively.

**Table 2:** Descriptive statistics by slope and aspect categories.

Indicator	Two-year-old stems	Three-year-old stems	Slope 10°	Slope 25°	Slope 35°	Semi-sunny	Semi-shaded
Mean DBH	6.23±1.03	3.09 ± 0.91	4.13 ± 1.51	4.51 ± 1.86	4.64 ± 2.21	4.64 ± 2.21	4.31 ± 1.67
Mean H	10.84±2.26	5.67 ± 1.55	6.94 ± 2.25	8.21 ± 3.30	8.52 ± 3.89	8.52 ± 3.89	7.55 ± 2.83
Mean AGB	6.72±2.27	1.65 ± 0.93	3.23 ± 2.44	3.88 ± 3.25	4.35 ± 3.39	4.35 ± 3.39	3.54 ± 2.83
Mean Culm Biomass	4.83±1.71	1.07 ± 0.61	2.22 ± 1.69	2.73 ± 2.49	3.08 ± 2.52	3.08 ± 2.52	2.47 ± 2.10
Mean Branch Biomass	0.97±0.46	0.31 ± 0.31	0.48 ± 0.41	0.61 ± 0.56	0.69 ± 0.54	0.69 ± 0.54	0.54 ± 0.48
Mean Leaf Biomass	0.92±0.36	0.28 ± 0.15	0.53 ± 0.44	0.54 ± 0.36	0.58 ± 0.46	0.58 ± 0.46	0.53 ± 0.39

Semi-sunny:azimuth between 45° and 135°; semi-shaded slopes (azimuth between 225° and 315°).

Systematic trends in growth and biomass partitioning were also observed across slope gradients and between aspect classes. Mean DBH, height, and AGB generally increased with steeper slopes, accompanied by higher proportional allocation to culm and branch biomass and lower allocation to leaf biomass. In two-year-old stems, DBH, height, and AGB were 24.1%, 36.3%, and 42.5% higher on steeper slopes relative to gentle slopes, while culm allocation rose from 70% to 73%; similar but weaker trends occurred in three-year-old individuals. Stems on semi-sunny slopes exhibited 12.85% greater height and 7.66% greater DBH than those on semi-shaded slopes, along with 22.88% higher AGB and correspondingly greater component biomasses. Although these trends align with previously reported patterns under contrasting light availability, the observational nature of this study precludes causal interpretation; all differences are therefore reported as empirical associations rather than statistically inferred effects.

### 3.2 Independent biomass models for components and aboveground biomass

**Figure 6:**
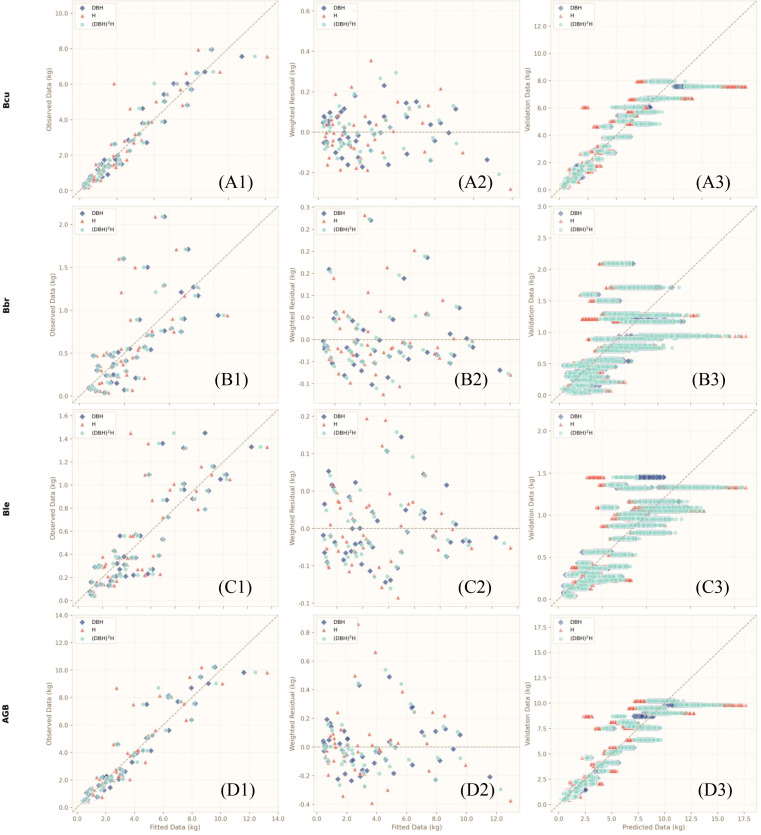
Plots of selected independent models for aboveground biomass (AGB) and its components with different predictors. Left **(A1, B1, C1, D1)**: bootstrap dataset vs. fitted biomass; Middle **(A2, B2, C2, D2)**: weighted residuals vs. fitted biomass; Right **(A3, B3, C3, D3)**: validation data (randomly split from the OOB dataset) vs. predicted biomass. Bcu, Bbr, Ble, and AGB represent the biomass of culm, branches, leaves, and total aboveground biomass, respectively.

We evaluated the modeling efficacy of three input variables: DBH, H, and D²H, across four categories of biomass components (culm, branch, leaf, and aboveground biomass [AGB]). Model predictors selection was performed via bootstrap resampling and AICc-based comparison, with three candidate predictors (DBH, H, (DBH)²H) evaluated across all biomass components (culm, branch, leaf, AGB). As shown in **Table 3**, the single-factor DBH power-law model consistently outperformed the H and (DBH)²H models, achieving the lowest AICc, highest bootstrap-R², and smallest bootstrap-RMSE/MAE for every component. Visual diagnostics (**Figure 6**) further confirmed that the DBH model exhibited tight alignment between fitted and observed values, stable random residuals without heteroscedasticity, and robust generalization, establishing it as the optimal, parsimonious predictor for subsequent biomass model development.

**Table 3:** Candidate models and associated AICc and bootstrap-based performance metrics.

Biomass type	Predictor	Model form	AICc	ΔAICc	Bootstrap-R²	Bootstrap-RMSE(Kg)	Bootstrap-MAE(Kg)
Culm Biomass	DBH	a·DBH^b	**-58.418**	0.000	0.949	0.499	0.371
	H	a·H^b	-3.116	55.302	0.822	0.928	0.557
	(DBH)²H	a·((DBH)²H)^b	-40.749	17.669	0.923	0.610	0.409
Branch Biomass	DBH	a·DBH^b	**-93.089**	0.000	0.529	0.340	0.227
	H	a·H^b	-81.971	11.118	0.396	0.384	0.252
	(DBH)²H	a·((DBH)²H)^b	-89.903	3.186	0.493	0.352	0.241
Leaf Biomass	DBH	a·DBH^b	**-138.380**	0.000	0.746	0.205	0.151
	H	a·H^b	-115.079	23.301	0.573	0.266	0.180
	(DBH)²H	a·((DBH)²H)^b	-130.329	8.051	0.695	0.224	0.164
AGB	DBH	a·DBH^b	**-12.504**	0.000	0.922	0.831	0.590
	H	a·H^b	34.674	47.178	0.774	1.411	0.825
	(DBH)²H	a·((DBH)²H)^b	3.312	15.816	0.888	0.993	0.641

Models are sorted by predictor order (DBH, H, (DBH)²H) within each component. Optimal models (lowest AICc) are shown in bold.

The final independent biomass models are shown in Equations 13–16:

(13) $$B_{cu}=a\cdot DBH^{b}$$

(14) $$B_{br}=a\cdot DBH^{b}$$

(15) $$B_{le}=a\cdot DBH^{b}$$

(16) $$AGB=a\cdot DBH^{b}$$

Using weighted nonlinear least squares, we fitted the independent DBH-based allometric models to each component of the bamboo biomass. **Table 4** shows the estimates of the parameters and the goodness-of-fit statistics. The culm biomass model had the best predictive accuracy of all the component, with an adjusted coefficient of determination (Adj.R²) of 0.946 and a root mean square error (RMSE) of 0.498. The values for a and b were a = 0.089 (95% CI: 0.123-0.220) and b = 2.161 (95% CI: 1.845-2.174). The aboveground biomass (AGB) model, which was fitted separately, performed well in the predictions, with Adj.R² = 0.918 and RMSE = 0.830. The estimates for the parameters were a = 0.170 (95% CI: 0.027-0.067) and b = 1.987 (95% CI: 1.381-1.929). The leaf biomass model had average fit statistics, with Adj.R² = 0.734 and RMSE = 0.205. The estimated values for the leaves were a = 0.044 (95% CI: 0.023-0.089) and b = 1.642 (95% CI: 1.256-2.071). The model for branch biomass gave an Adj.R² of 0.508 and an RMSE of 0.339. The parameter estimates were a = 0.050 (95% CI: 0.066–0.113) and b = 1.609 (95% CI: 2.019–2.334).

**Table 4:** Parameter estimates and 95% confidence intervals of the independent models.

Model form	a	b	a CI [lower, upper]	b CI [lower, upper]	RMSE	Adj.R^2^
$B_{cu}=a\cdot DBH^{b}$	0.089	2.161	[0.123, 0.220]	[1.845, 2.174]	0.498	0.946
$B_{br}=a\cdot DBH^{b}$	0.050	1.609	[0.066, 0.113]	[2.019, 2.334]	0.339	0.508
$B_{le}=a\cdot DBH^{b}$	0.044	1.642	[0.023, 0.089]	[1.256, 2.071]	0.205	0.734
$AGB=a\cdot DBH^{b}$	0.170	1.987	[0.027, 0.067]	[1.381, 1.929]	0.830	0.918

All parameters (a, b) and 95% confidence intervals (CIs) were estimated using the entire dataset. Bcu, Bbr, Ble, and AGB represent culm biomass, branch biomass, leaf biomass, and aboveground biomass, respectively. All models were fitted as independent allometric equations with DBH as the sole predictor.

The confidence intervals for the parameters varied in width across components. Notably, the culm model produced the narrowest confidence intervals for the exponent parameter *b*, while the branch model exhibited wider intervals, reflecting greater uncertainty in branch biomass estimation.

### 3.3 Compatible biomass model system based on seemingly unrelated regression

According to the research, when the compatible biomass model system adopts the same predictors as those of the independent models, it helps to improve the accuracy and stability of the model, ensuring the consistency and scientific validity of the biomass estimation (Dong et al., 2014; Fu et al., 2018).Therefore, in this study, the compatible model also adopted the same predictor as the independent biomass model, that is, using the diameter at breast height (DBH) as the sole predictor.

The compatible model system (Seemingly Unrelated Regression) is shown in Equations 17–20:

(17) $$B_{cu}=a_{1}\cdot DBH^{b_{1}}$$

(18) $$B_{br}=a_{2}\cdot DBH^{b_{2}}$$

(19) $$B_{le}=a_{3}\cdot DBH^{b_{3}}$$

(20) $$AGB=B_{cu}+\;B_{br}+B_{le}=a_{1}\cdot DBH^{b_{1}}+a_{2}\cdot DBH^{b_{2}}+a_{3}\cdot DBH^{b_{3}}$$

The compatible biomass models were adjusted utilizing the nonlinear seemingly unrelated regression (SUR) methodology with direct total control. **Table 5** shows the parameter estimates and goodness-of-fit statistics for the components’ biomass. The culm biomass model had the best predictive power of the three components, with an Adj.R² of 0.946 and an RMSE of 0.498. The values for a and b for culms were a = 0.088 (95% CI: 0.066-0.111) and b = 2.163 (95% CI: 2.028-2.335). The aboveground biomass (AGB) model, which was based on the sum of the three component predictions under the additivity constraint, had good predictive power, with an Adj.R² of 0.909 and an RMSE of 0.834. The parameter estimates for the total model were the same as those for the component models, which shows that the system is naturally compatible. The leaf biomass model had average fit statistics, with Adj.R² = 0.734 and RMSE = 0.205. The values for the leaf model were a = 0.044 (95% CI: 0.026–0.066) and b = 1.640 (95% CI: 1.381–1.927). The model for branch biomass showed poor performance, with an adjusted R² of 0.508 and an RMSE of 0.339. The parameter estimates were a = 0.049 (95% CI: 0.025–0.091) and b = 1.616 (95% CI: 1.259–2.026).

**Table 5:** Parameter estimates and 95% confidence intervals of the compatible models.

Model form	a	b	a CI [lower, upper]	b CI [lower, upper]	RMSE	Adj.R^2^
$B_{cu}=a_{1}\cdot DBH^{b_{1}}$	0.088	2.163	[0.066, 0.111]	[2.028, 2.335]	0.498	0.946
$B_{br}=a_{2}\cdot DBH^{b_{2}}$	0.049	1.616	[0.025, 0.091]	[1.259, 2.026]	0.339	0.508
$B_{le}=a_{3}\cdot DBH^{b_{3}}$	0.044	1.640	[0.026, 0.066]	[1.381, 1.927]	0.205	0.734
$AGB=B_{cu}+B_{br}+B_{le}=a_{1}\cdot DBH^{b_{1}}+a_{2}\cdot DBH^{b_{2}}+a_{3}\cdot DBH^{b_{3}}$	0.088 0.049 0.044	2.163 1.616 1.640	idem	idem	0.834	0.909

All parameters (a, b) and 95% confidence intervals (CIs) were estimated using the entire dataset. Bcu, Bbr, Ble, and AGB represent culm biomass, branch biomass, leaf biomass, and aboveground biomass, respectively. All models were fitted as compatible allometric equations with DBH as the sole predictor.

The 95% confidence intervals for all parameters were fairly small, which means that the estimates were fairly accurate. The additivity constraint ( $\hat{AGB}=\;\hat{B_{cu}}+\;\hat{B_{br}}\;+\hat{B_{le}}$) was strictly enforced through the direct total control method, which made sure that the predictions for the biomass components were biologically consistent.

### 3.4 Performance comparison of independent vs. compatible biomass models

**Figure 7:**
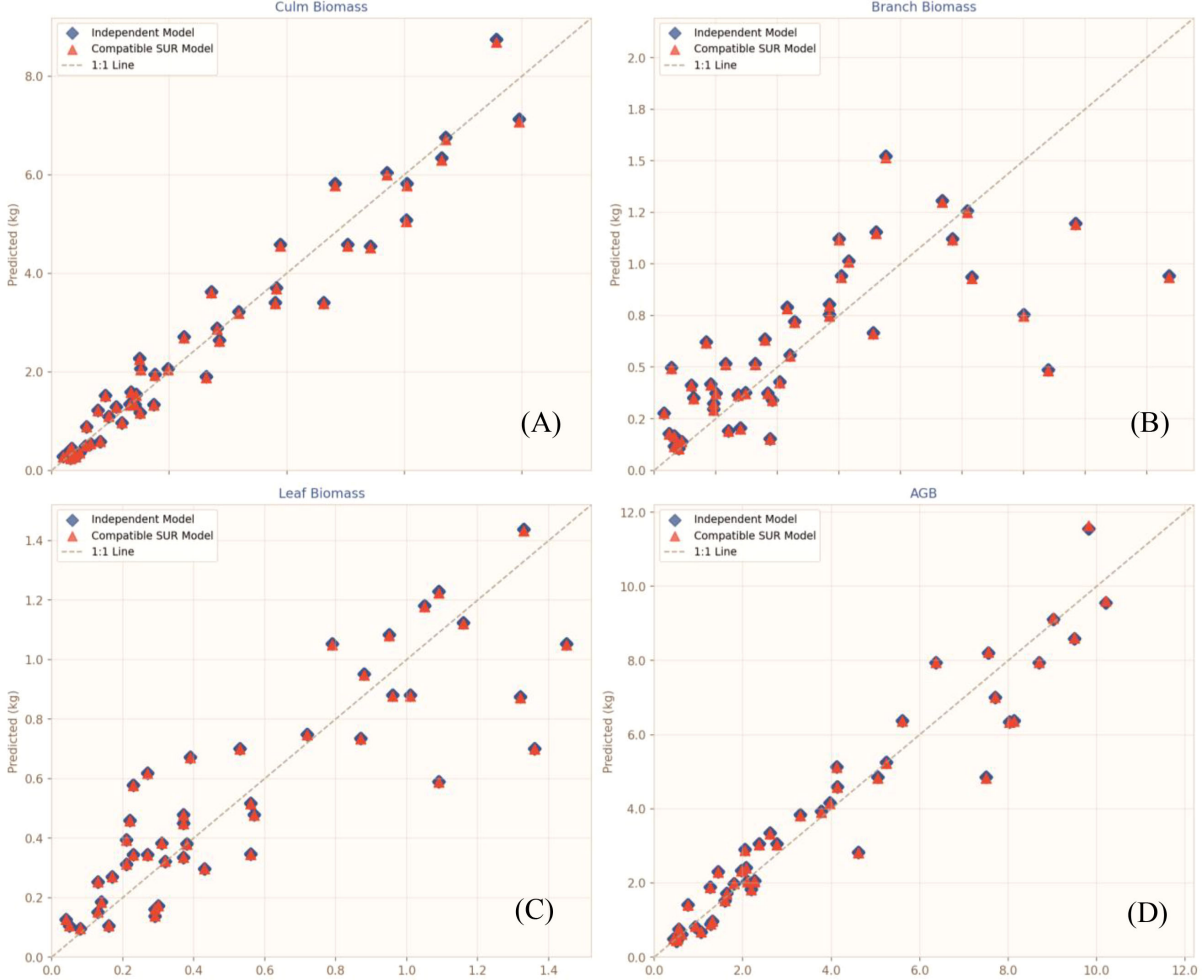
Accuracy of independent vs. compatible model fitting for different biomass components. **(A)** Culm biomass; **(B)** Branch biomass; **(C)** Leaf biomass; **(D)** Aboveground biomass.

**Figure 8:**
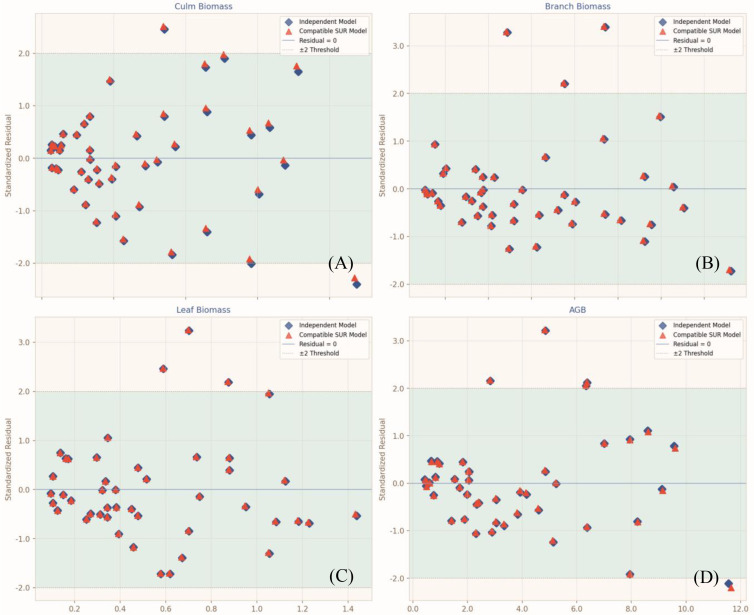
Comparison of standardized residuals from independent vs. compatible models by component. **(A)** Culm biomass; **(B)** Branch biomass; **(C)** Leaf biomass; **(D)** Aboveground biomass.

Predictive performance of the independent biomass models and the compatible SUR model system was assessed using 1,000 bootstrap iterations with out-of-bag (OOB) predictions to avoid overestimation of model fitting performance. Key evaluation metrics, including Bootstrap-R², RMSE, MAE, and relative bias, are summarized in **Table 6**, while model fitting accuracy and residual patterns are illustrated in **Figures 7**, **8**, respectively.

**Table 6:** Bootstrap-derived predictive performance of the compatible and independent models.

Biomass component	Model type	Bootstrap-R²	RMSE	Bias (%)	MAE (kg)
Culm	Compatible (SUR)	0.944 ± 0.021	0.491 ± 0.080	4.151 ± 5.146	0.371 ± 0.069
Branch	Compatible (SUR)	0.513 ± 0.155	0.328 ± 0.085	48.258 ± 26.708	0.225 ± 0.051
Leaf	Compatible (SUR)	0.734 ± 0.090	0.199 ± 0.040	20.847 ± 11.345	0.150 ± 0.028
AGB	Compatible (SUR)	0.915 ± 0.036	0.810 ± 0.168	4.470 ± 5.045	0.581 ± 0.122
Culm	Independent	0.944 ± 0.022	0.492 ± 0.081	5.041 ± 5.193	0.373 ± 0.069
Branch	Independent	0.512 ± 0.155	0.328± 0.084	49.861 ± 27.028	0.227 ± 0.051
Leaf	Independent	0.733 ± 0.090	0.199 ± 0.039	21.177 ± 11.367	0.150 ± 0.027
AGB	Independent	0.916 ± 0.036	0.807 ± 0.164	4.570 ± 5.087	0.584 ± 0.120

Values are presented as mean ± standard deviation derived from 1,000 bootstrap iterations using out-of-bag predictions. RMSE and MAE are expressed in kilograms, and bias is expressed as percentage of observed values. Bootstrap-based metrics reflect predictive rather than fitting performance.

For culm biomass, both modeling approaches achieved similarly high predictive accuracy, with identical Bootstrap-R² values of 0.944 and comparable RMSE and MAE. The compatible model exhibited slightly lower relative bias (4.151 ± 5.146%) than the independent model (5.041 ± 5.193%). Observed versus predicted values were tightly distributed along the 1:1 line (**Figure 7A**), and standardized residuals were randomly scattered around zero with no obvious heteroscedasticity (**Figure 8A**), indicating high stability and reliability for culm biomass estimation. For aboveground biomass (AGB), both models performed robustly, with Bootstrap-R² values above 0.91 and relative bias below 5%. Predictions were closely aligned with observations (**Figure 7D**), and residuals were uniformly distributed across the fitted range (**Figure 8D**), confirming strong predictive capacity for total aboveground biomass.

By contrast, prediction performance was considerably lower for branch and leaf biomass. Branch biomass models yielded the lowest Bootstrap-R² (approximately 0.51) and the highest relative bias (exceeding 48%), whereas leaf biomass models showed moderate accuracy with Bootstrap-R² around 0.73 and bias near 21%. Predictions for these components were more scattered (**Figures 7B, C**), though residual patterns remained random without systematic bias (**Figures 8B, C**), suggesting that prediction uncertainty mainly reflects inherent variability in branch and leaf growth rather than structural model deficiencies.

Overall, the compatible SUR system performed marginally better than independent models across most components, with slightly lower RMSE and relative bias. More importantly, the SUR framework inherently ensured additivity among component biomass estimates, eliminating the inconsistencies between summed component predictions and total AGB that often arise from independently fitted models. Visual comparisons confirmed that predicted values and residual distributions of the two modeling strategies were highly consistent, indicating that compatibility was achieved without compromising predictive accuracy.

The bootstrap-OOB framework employed here provided a robust assessment of model generalization rather than mere fitting performance. The mean and standard deviation of the metrics across 1,000 iterations further quantified estimation uncertainty, supporting the reliability of the developed models for practical application in biomass and carbon stock assessment.

## 4 Discussion

Sanya has a tropical monsoon climate that is significantly moderated by the ocean. The climate is characterized by warm, humid conditions with abundant rainfall and minimal annual temperature variation (Wang et al., 2019; Zhou and Shen, 2018). Although cold spells are occasionally experienced, temperatures remain well above the 18 °C tropical threshold (Wang et al., 2020). The high annual temperatures and long frost-free period align well with the ideal growth requirements of *D. brandisii*, contrasting with the cooler, frost-prone subtropical regions of Yunnan and Guangxi (Tao et al., 2024; Wang et al., 2015; Tang, 2011). The hot, humid climate affects plant growth rates and water use efficiency, influencing growth patterns and distribution (Li et al., 2021). This makes it a unique location in which to study the species’ adaptability to a hot, humid, ocean-influenced climate.

Previous studies have demonstrated that in various bamboo species, including *Phyllostachys edulis* cv. Pubescens, *Bambusa rigida*, *Bambusa gibba*, *Gigantochloa apus*, and others, culm biomass consistently accounts for the largest proportion of total aboveground biomass (AGB), followed by branches, with leaves providing the lowest contribution (Wang, 2014; Zheng, 2014; Sujarwo, 2016). We found a similar pattern of biomass allocation for *D. brandisii* cultivated in Sanya, namely: culm > branch > leaf, further confirming the dominance of the structural components in aboveground biomass partitioning across diverse bamboo taxa (Jyoti Nath et al., 2009). This may be due to strategies for rapid vertical growth to compete for light or facilitate nutrient transport (Wang et al., 2021b; Huang et al., 2020b). Clumping bamboo spreads outward from the central mother stem, so younger bamboo stems occupy the cluster’s periphery and thereby gain preferential access to sunlight, water, and nutrients (Yao et al., 2023). They also benefit from developing from a larger, more mature rhizome. *D. brandisii* growing in Yunnan allocated a substantially greater proportion of biomass to culms (82–87%) than in Sanya (67%), with the allocation ratios to branches and leaves increasing with stem age (Wang et al., 2025). This suggests that in different climatic regions, *D. brandisii* may adapt to local environmental conditions by adjusting the proportional allocation of biomass among culms, branches, and leaves.

Consistent with other findings on sympodial bamboos (Zheng, 2016), all clump expansion metrics, stem number per clump, mean DBH and height of new stems, and clump width, increased year-on-year. In a young bamboo forest, the component biomass and aboveground biomass of newly emerged stems (2-year-old) are generally higher than those of older stems (3-year-old). This is consistent across several species, including *Pleioblastus amarus*, *D. asper*, and *D. brandisii* (Zuo et al., 2024; Yang, 2015). For instance, in the present study area, the first year (2021) was the establishment year for the plantation; the mother plant was in an acclimation period with underdeveloped root systems and weak nutrient uptake, resulting in relatively small stems that emerged that year (i.e., the three-year-old stems in 2024). In the following year (2022), after the mother plant’s root systems had become well established and the bamboo stand had entered the early stage of closed canopy formation, the stems that emerged (i.e., the two-year-old stems) received better nutrition and thus grew larger (Christanty, 1989; Castañeda-Mendoza et al., 2012).This outcome can be attributed to differences in growing conditions resulting from stem emergence in different years (i.e., different stages of stand establishment), rather than to culm shrinkage due to aging. With advancing age, the proportion of biomass allocated to culms decreases, with more biomass being allocated to branches and leaves (Hu et al., 2025; Wang et al., 2025). The sympodial clumping structure of *D. brandisii* means that newer stems are closer to the source of nutrients, have access to more peripheral light, and are generally more vigorous than the older stems toward the center of the clump (Shi et al., 2024; Ma, 2008).

Some studies have demonstrated that slope and aspect affect bamboo’s aboveground biomass and how it is distributed among the culm, branches, and leaves (Zhou et al., 2023; Bahru and Ding, 2020). However, research on aboveground biomass allocation of *D. brandisii* has shown that there were no statistically significant differences across slope gradients and aspects (Urgilés Ortiz, 2019; Xu, 2008). This is usually because soil moisture and nutrient availability are the primary factors influencing biomass formation, while the effect of topography is easily masked by differences in site conditions (Wang et al., 2011; Zang et al., 2022), particularly in tropical/sub-tropical regions where the moderating effect of aspect on temperature and humidity is limited (Lu et al., 2016). The ifluence of slope and aspect can affect light availability which in turn can change photosynthetic performance and the way aboveground biomass is divided. Semi-sunny to moderately shaded environments are often best for growth compared to full shade or too much direct light (Park et al., 2023). However, the differences in light exposure among different slope aspects can be compensated through plant morphological plasticity (such as canopy structure adjustment), thereby weakening the influence of slope aspect on the biomass allocation at the organ level (Gao et al., 2023). Although we found a link between above-ground biomass and slope orientation as well as slope gradient, due to the limited number of experimental plots, we did not attempt to demonstrate the causal relationship between topographical factors and biomass. Future research can adopt control experiments or long-term fixed-point monitoring methods to clarify the potential regulatory mechanisms.

The variables influencing the development of biomass models include growth factors such as DBH, H, (DBH)²H, and age, as well as environmental factors such as longitude and latitude, slope and slope direction, temperature, and sunlight (Juan et al., 2024; Wang et al., 2021a; Wi et al., 2017; Huy et al., 2019b; Chantarat et al., 2023). Although adding more predictors to a model may theoretically improve its accuracy, it can lead to non-convergence and parameter estimation bias problems caused by over-parameterization (Fu et al., 2013, Fu et al., 2017; Zhou et al., 2021, Zhou et al., 2023). In our study, topographic factors and age were not included in the models due to the limitations of the experimental site. This not only simplified the model structure but also avoided potential multicollinearity issues that might arise from incorporating these factors with other candidate predictors (Zhou et al., 2025). According to model comparisons, DBH-based models exhibited the optimal goodness of fit, lowest AICc and best bootstrap validation performance for all biomass components and total aboveground biomass (AGB), whereas models using H or (DBH)²H showed relatively higher AICc, larger prediction errors and lower explanatory power. Meanwhile, diagnostic plots further verified that DBH-only models presented randomly distributed residuals and satisfactory consistency between fitted and observed values, suggesting robust reliability and absence of obvious systematic bias. As the most accessible and accurately measurable variable in field surveys, DBH greatly reduces measurement difficulty and cost compared with H, which is difficult to determine with high precision (Skladan et al., 2025; Xu et al., 2025). Moreover, the significant positive correlation between DBH and H implies that introducing both variables would aggravate multicollinearity and reduce model stability (Li et al., 2025). Additionally, previous studies have shown that including age as an additional covariate contributes little to model accuracy (Li et al., 2024b), which further supports our decision to exclude age from the models. Therefore, DBH alone is sufficient to capture most variation in biomass accumulation, and the marginal improvement from adding other predictors is not enough to offset the increased model complexity and operational inconvenience. These results are consistent with numerous previous studies that have recommended DBH as the primary and most practical predictor for biomass modeling (Liang et al., 2022; Huang, 2020; Vuorinne et al., 2021; Melo et al., 2015; Siddique et al., 2021; Ren, 2025).

Among the various biomass models for individual plant components and aboveground biomass (AGB), whether independent or compatible, single-variable, binary-variable, or multivariate, the culm biomass (Bcu) and AGB models demonstrated the highest fitting accuracy within their respective categories. The culm model slightly outperformed the AGB model (both with R² values exceeding 0.9). These were followed by the leaf biomass models, which showed slightly lower accuracy (generally with R² > 0.7). The branch biomass models exhibited the lowest fitting accuracy, with all models yielding R² values around 0.5, a phenomenon that has been widely reported (Melo et al., 2015; Wang et al., 2021c; Zheng, 2014; Zhou et al., 2022). Branch biomass models do not make very accurate predictions, as reflected by the low explanatory power (R² ~ 0.51) and high bias (~48–50%), which could be because the growth length of branches varies substantially (due to factors such as micro-environment, light competition, nutrient availability, and others, and DBH is difficult to fully capture). As modeling individual components separately compromises the additivity of the total biomass model, recent biomass studies have increasingly accounted for additivity and inter-component correlations in biomass estimation (e.g., Dong et al., 2016; Zhou et al., 2023). As the primary methods for estimating additive systems, Seemingly Unrelated Regression (SUR) and Two-Stage Error Structure Modeling (TSEM) are widely used simultaneously to fit component-specific biomass models (Dong et al., 2020; Zhou et al., 2022, Zhou et al., 2025). In this study, we applied the SUR approach to construct a compatible additive model system for the aboveground biomass of *D. brandisii*. The SUR simultaneously estimates parameters by solving a system of equations and imposes mathematical constraints (e.g., the parameters of the total model are derived from those of the component models) to ensure that the sum of the component predictions exactly equals the total prediction (Parresol, 1999, Parresol, 2001; Cao et al., 2019; Chen et al., 2020). Zhao et al., 2019b) further verified that SUR, while guaranteeing additivity, can improve the efficiency of parameter estimation through its error-covariance structure. The compatible model provides a ready-to-use tool for forest-carbon monitoring, especially when both component and total biomass must be reported simultaneously (Zhuo et al., 2016). Adopting such models in national forest inventories avoids “carbon-accounting gaps” and ensures consistency of estimates across scales (Grimm et al., 2006; Jenkins et al., 2003; Shannon et al., 2025). It should be noted that the compatibility or additive nature of the SUR model cannot compensate for the branch biomass model’s deficiency in predicting branch biomass. Therefore, in the future, considering the addition of predictor variables (such as branch length, canopy width and length) will be crucial for improving the accuracy of predicting branch biomass.

## 5 Conclusions

This study is the first to empirically assess the aboveground biomass allocation characteristics of *D. brandisii* in the tropical monsoon climate of Sanya and to establish an individual-plant-scale aboveground biomass model. The results revealed a stable biomass dominance hierarchy (culm > branches ≈ leaves) with an average ratio of 4:1:1. Compared with a *D. brandisii* population planted in Yunnan, the culm allocation ratio in Sanya was significantly lower (67%), indicating that this species exhibits phenotypic plasticity in warm, humid, ocean-moderated environments. An “age-size” reversal phenomenon was observed: the diameter at breast height (DBH), height, and total biomass of two-year-old stems were generally significantly greater than those of three-year-old stems. This pattern is likely attributable to the gradual maturation of the mother bamboo plant, including its rhizome system, during the early stage of stand establishment. Using seemingly unrelated regression (SUR) with DBH as the sole predictor variable, we developed the first compatible biomass model system for this species. Although slope and aspect were associated with biomass trends, causal inference remains precluded due to observational constraints and limited plot replication, which highlights the necessity for controlled experiments to disentangle topographic effects. The SUR system ensures that the biomass of components can be aggregated, eliminates discrepancies in predictions, and provides a reliable, biologically valid method for assessing carbon stock in tropical bamboo forests. Future research should focus on the effects of light, temperature, and water availability on the plasticity of biomass allocation in *D. brandisii* and on the incorporation of architectural predictors to improve branch biomass estimation in heterogeneous topographic environments.

## Data Availability

The original contributions presented in the study are included in the article/**Supplementary Material**. Further inquiries can be directed to the corresponding authors.
